# A Pathogenic Variant p.Phe177Val in *PSEN1* Causes Early-Onset Alzheimer’s Disease in a Chinese Family

**DOI:** 10.3389/fgene.2020.00713

**Published:** 2020-07-10

**Authors:** Bin Jiang, Min Bi, Jun Li, Qi Liu, Nai-An Xiao, Jie Fang, Man-Yi Shi, Zi-Wen Yu, Qi-Lin Ma, Sui-Jun Tong, Kun-Mu Zheng

**Affiliations:** Department of Neurology, First Affiliated Hospital, Xiamen University, Xiamen, China

**Keywords:** Alzheimer’s disease, *PSEN1*, pathogenic variant, whole-exome sequencing, Chinese family

## Abstract

Familial Alzheimer’s disease (FAD) present as a positive family history of cognitive decline, with early onset and an autosomal dominant inheritance pattern. FAD is mainly caused by the mutations in the genes encoding for amyloid precursor protein (*APP*), presenilin-1 (*PSEN1*), and presenilin-2 (*PSEN2*). In the present study, we identified a variant (c.529T > G, p.Phe177Val) in *PSEN1* across three generations in a Chinese family with FAD using whole-exome sequencing. The mean age of onset was 39 years (range: 37 to 40 years) in this family. In cell transfection studies, the mutant PSEN1 protein carrying p.Phe177Val increased both the production of Aβ42 and the ratio of Aβ42 over Aβ40, as compared to wild-type PSEN1. Our results confirm the pathogenicity of *PSEN1* p.Phe177Val variant in FAD and broaden the clinical phenotype spectrum of FAD patients with *PSEN1* p.Phe177Val variant.

## Introduction

Alzheimer’s disease (AD) is the most common form of dementia and is characterized by a progressive decline in memory and cognition. One major pathological hallmark of AD is senile plaques composed of amyloid-β (Aβ) peptides (Hardy and Selkoe, 2002). Cases with familial aggregations are defined as familial AD (FAD), which accounts for less than 1% of all AD cases (Bekris et al., 2010). Patients with FAD typically have autosomal dominant inheritance and are commonly with an age of onset before 65 years (Hardy, 2006). FAD is mainly caused by mutations in the genes encoding for amyloid precursor protein (*APP*), presenilin 1 (*PSEN1*), and presenilin 2 (*PSEN2*; Tanzi, 2012). According to the Human Gene Mutation Database (HGMD), 286 mutations within *PSEN1*, 78 mutations within *APP*, and 37 mutations within *PSEN2* have been associated with AD.

Presenilin-1 is the leading causative gene of FAD. It is located on chromosome 14, with 12 exons encoding for a protein with 9 transmembrane helices (Spasic et al., 2006). *PSEN1* protein is the major component of the γ-secretase complex, which cleaves APP. Several *PSEN1* mutations lead to functional change in the γ-secretase may result in AD. In previous studies, *PSEN1* mutations have increased the production of Aβ42 and the ratio of Aβ42 over Aβ40 in cell lines and transgenic animals, indicating that these mutations have a pathogenic effect in FAD (Borchelt et al., 1996; Mehta et al., 1998). In the present study, using whole-exome sequencing (WES), we detected a variant of the *PSEN1* gene across three generations in a Han Chinese family with FAD. We then investigated the pathogenicity of this variant using functional studies.

## Materials and Methods

### Subjects

The proband with AD symptoms and seven family members were recruited for the study in First Affiliated Hospital of Xiamen University. The subjects were evaluated by at least two senior neurologists. The criteria of the DSM-IV-TR (American Psychiatric Association, 2000) and NINCDS-ADRDA (McKhann et al., 1984) were applied to diagnose probable AD. The Mini-Mental State Examination (MMSE) and the activities of daily living (ADL) scales were used to evaluate the patients as in previous report (Tao et al., 2017). Five hundred healthy controls with similar ages and origins were included in the study. Written informed consent was obtained from each participant or from their guardians. Ethical approval was provided by the Research Committee of First Affiliated Hospital of Xiamen University.

### Genetic Testing

Genomic DNA was extracted from an EDTA-treated sample of the patients’ peripheral blood using the Blood Genomic DNA Extraction Kit (Qiagen, Germany). Apolipoprotein E (*APOE*) genotypes were determined by multiplex amplification refractory mutation system polymerase chain reaction (Jiang et al., 2018). The DNA sample of the proband was processed using WES. The details of the WES protocols and bioinformatic analysis have been described in previous publications (Li et al., 2017; Jiang et al., 2019). In brief, an enriched DNA sample was sequenced on the Illumina Nova 6000 platform (Kangso Medical Inspection Co. Ltd., Beijing, China). All the variants were annotated using ANNOVAR software. The frequency of the variants in the general population was identified by the 1000 Genomes Project^1^, the genome aggregation database^2^, the ExAC database^3^, and the single-nucleotide polymorphism database^4^. SIFT^5^, CADD^6^, and PolyPhen-2^7^ were used to predict the possible functional changes caused by the variants. The potential variants were verified by Sanger sequencing. All the available family members, as well as the 500 healthy individuals, were sequenced to validate the confirmed variants.

### Cell Culture and Transfection

SH-SY5Y cells, which stably over-express the APP751 isoform, were cultured in Dulbecco’s modified Eagle’s medium (GIBCO, United States) supplemented with 10% fetal bovine serum (GIBCO, United States). The coding sequence of the human *PSEN1* gene (NM_000021.3) was cloned into a pFLAG-CMV4 vector. F177V mutant plasmid was created using PCR mutagenesis and validated by Sanger sequencing. Cells were transiently transfected with the *PSEN1* wild-type (WT) plasmid, the F177V mutant plasmid, or the empty vector using Lipofectamine3000 (Invitrogen, United States) according to the manufacturer’s protocols.

### Functional Studies in Cultured Cells

Protein expression levels were determined using Western blot analysis. The protein samples were resolved by 10% SDS-PAGE and transferred to polyvinylidene fluoride membranes (BIO-RAD, United States), which were then incubated overnight at 4°C in the following primary antibodies: anti-PS1 (Thermo Fisher Scientific, cat. no. PA5-98093, United States), and anti-GAPDH (CST, cat. no. 5174, United States). Three independent experiments that included both technical and biological replicates were performed. To explore Aβ levels, Aβ40, and Aβ42 ELISA kits (Invitrogen, United States) were used according to the manufacturer’s instructions. In brief, the cell media were mixed with Aβ 40-, or Aβ42-specific antibody and shake-incubated in a 96-well plate overnight at 4°C. After incubation with secondary antibody for 30 min, the reaction with substrate was carried out at room temperature. The color intensity was measured at 450 nm using a multimode plate reader (EnVision^®^, Perkin Elmer, United States). Five independent experiments that included both technical and biological replicates were performed.

### Statistical Analysis

Data were presented as mean ± standard error of mean. Statistical significance was analyzed using one-way ANOVA on GraphPad Prism 5 software. Differences were considered statistically significant at *P*-values < 0.05.

## Results

### Identification of the Variant

**FIGURE 1:**
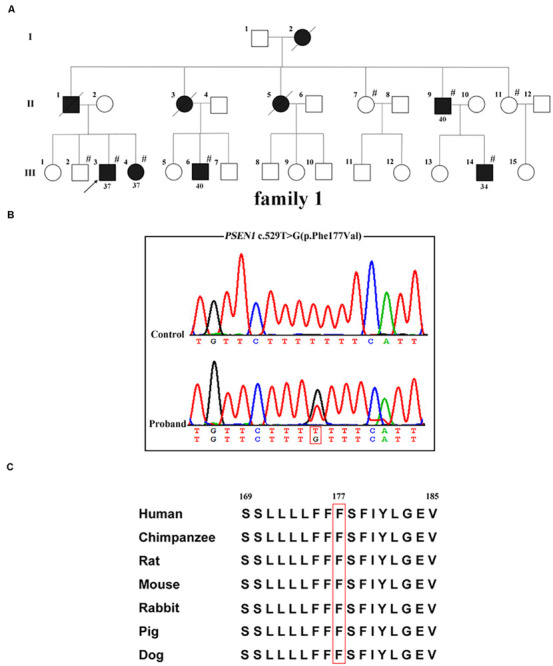
*PSEN1* variant identified in patients with FAD. **(A)** Pedigrees of the family with FAD carrying the p.Phe177Val variant in *PSEN1*. Arrows indicate the proband of the family. Hash symbols indicate individuals who were recruited for mutation analysis of *PSEN1*. Numbers indicate the age of onset. **(B)** Sequencing chromatograms of the c.529T > G (p.Phe177Val) variant in *PSEN1*. **(C)** Conservation analysis of the p.Phe177Val variant in *PSEN1* (amino acid position 177 boxed in red).

Whole-exome sequencing was carried out in the proband (family 1, III-3; Figure 1A); 97.4% of the target bases had >30x coverage. After filtering, a heterozygous missense variant *PSEN1* c.529T > G (p.Phe177Val) was detected. Sanger sequencing confirmed the presence of the variant (Figure 1B). It was absent from 1000 Genomes project, the ExAC database, the dbSNP, and the genome aggregation database. Furthermore, the variant was not detected in the 500 healthy controls, and the Phe177 residue was found to be highly conserved across different animal species (Figure 1C). Family co-segregation analysis showed that the variant occurred in patients with AD symptoms (family 1, II-9, III-4, III-6, and III-14) but not in the healthy members (family 1, II-7, II-11, and III-2). Furthermore, SIFT (score: 0.006), Polyphen2 (score: 0.992), and CADD (score: 28.3) predicted that the variant would be deleterious.

### Functional Analysis and Classification of the Variant

**FIGURE 2:**
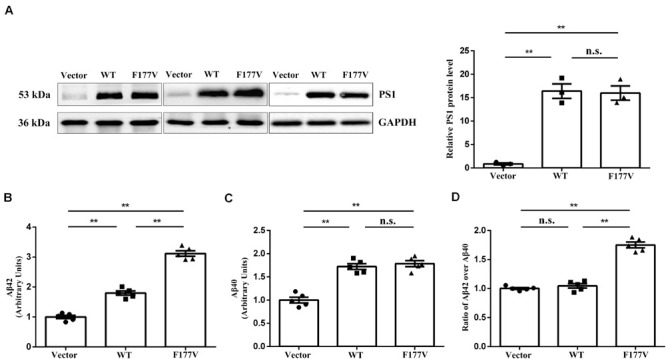
Transfection studies of wild-type (WT) and p.Phe177Val (F177V) mutant PS1 protein in cultured cells. **(A)** Western blot analysis of PS1 protein expression. The PS1 protein was detected using an anti-PS1 antibody. **(B,C)** The relative amounts of Aβ42 and Aβ40 in the media of transfected cells was determined by ELISA. **(D)** The ratios of Aβ42 over Aβ40 were calculated for comparison. Error bars indicate mean ± SEM, n.s. = not significant, **P < 0.01, and one-way ANOVA.

To explore the biological effect of the p.Phe177Val variant in *PSEN1*, we transiently transfected either WT, or F177V mutant plasmids into cell lines. No significant difference was observed between WT and mutant PSEN1 protein levels using Western blot analysis [F177V vs. WT, 15.99 ± 1.52 (95% confidence interval 9.47–22.51) vs. 16.40 ± 1.54 (95% confidence interval 9.78–23.03), *p* = 0.97; Figure 2A]. Furthermore, the mutant PSEN1 protein led to greater Aβ42 production than the WT protein [F177V vs. WT, 3.12 ± 0.09 (95% confidence interval 2.861–3.375) vs. 1.80 ± 0.07 (95% confidence interval 1.600–1.998), *p* < 0.01; Figure 2B]. However, the production of Aβ40 was comparable between the WT and mutant PSEN1 proteins [F177V vs. WT, 1.79 ± 0.06 (95% confidence interval 1.607–1.963) vs. 1.72 ± 0.06 (95% confidence interval 1.550–1.894), *p* = 0.76; Figure 2C]. Thus, the p.Phe177Val variant increased the ratio of Aβ42 over Aβ40 [F177V vs. WT, 1.75 ± 0.05 (95% confidence interval 1.612–1.891) vs. 1.05 ± 0.03 (95% confidence interval 0.943–1.150), *p* < 0.01; Figure 2D]. According to the American College of Medical Genetics and Genomics (ACMG) standards (Richards et al., 2015), the p.Phe177Val variant in *PSEN1* was classified as pathogenic, with one piece of strong evidence, two pieces of moderate evidence, and two pieces of supporting evidence. Specifically, the functional *in vitro* study indicating that the variant had a damaging effect was considered strong evidence of pathology. The absence of the variant in the 500 controls and in the databases was considered moderate evidence, as was the missense amino acid change occurring at the same position as another pathogenic missense change. The positive co-segregation in this family and the predictions of deleterious effect by computational software programs were considered supporting evidences.

### Clinical Features of Patients Carrying the Variant

**FIGURE 3:**
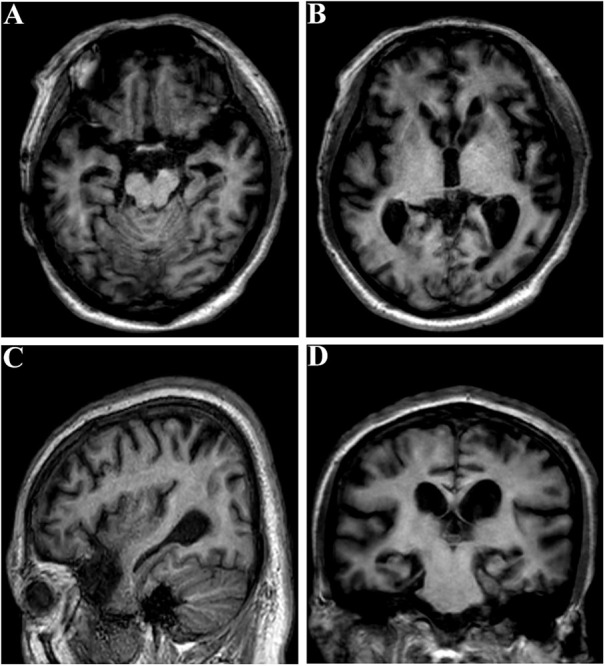
Brain MRI of the proband (family 1, III-3) at the age of 43. **(A–C)** Global brain atrophy especially in the temporal region and hippocampus. **(D)** Widened choroid fissure and temporal horn dilation, hippocampal atrophy. The scale for median temporal lobe atrophy (MTA-scale) was 2–3 degree.

The proband (family 1, III-3) developed memory deficits as the first symptom at the age of 37. He had difficulties with calculation and showed disorientation in new surroundings, as well as a progressive decline in memory. At the age of 40, these cognitive disturbances had caused major difficulties at work (he worked as a driver). The patient developed neuropsychiatric symptoms, such as depression and anxiety, when he was 42. Myoclonus, extrapyramidal signs, spastic paraparesis, and cerebellar ataxia were not observed. The patient’s MMSE score was 4/30 and his ADLs were impaired (ADL score = 60) when he consulted our memory clinic at the age of 43. His *APOE* genotype was ε3/ε3. Brain magnetic resonance imaging (MRI) analysis revealed global brain atrophy, especially in the temporal region and hippocampus (Figure 3). He reported a family history of dementia. His younger sister (family 1, III-4), who carried the same variant, displayed amnesia as her first symptom at the age of 37. She could still work as a cleaner in the hotel when referred to our memory clinic at the age of 40. Her MMSE score was 23/30 and her ADL score was 20. Her *APOE* genotype was ε3/ε3. She refused the brain MRI scan.

Another patient (family 1, II-9) exhibited behavioral changes and anxiety as his first presentations at the age of 40. He gradually developed deficits in memory, which grew progressively worse. Relatives informed us that he was impaired in both work (he worked as a waiter in the restaurant) and daily life after his cognitive disturbances had progressed. No atypical phenotypes were observed, such as myoclonus, spastic paraparesis, or cerebellar ataxia. He is currently cared for permanently by his wife at the age of 58. Another patient (family 1, III-6) displayed anxiety and irritation starting at the age of 40. He was admitted to the Xiamen municipal mental health center and treated with antipsychotic drugs, which controlled the symptoms well. He did not report memory problems when he consulted our hospital at the age of 42. Another patient (family 1, III-14) developed anxiety as the first symptom when he was 34. His relatives reported that he had become impatient and irritated. No memory deficits were observed when he was 35. All of these patients (family 1, II-9, III-6, and III-14) refused further examinations, such as brain MRI scan, MMSE test, and ADL test. The clinical features of the patients carrying the *PSEN1* p.Phe177Val variant are summarized in Table 1.

**TABLE 1:** Clinical data of patients carrying the *PSEN1* p.Phe177Val variant.

**Patient No.**	**III–3 (proband)**	**III–4**	**II–9**	**III–6**	**III–14**
Sex	Male	Female	Male	Male	Male
Age at onset (years)	37	37	40	40	34
Age at study (years)	43	40	58	42	35
*APOE* genotype	ε3/ε3	ε3/ε3	ε2/ε3	ε3/ε3	ε2/ε3
MMSE	4	23	NA	NA	NA
ADL	60	20	NA	NA	NA
Onset symptom	Amnesia	Amnesia	NPS	NPS	NPS
Memory deficits	+	+	+	–	–
Other cognitive symptoms	Disorientation dyscalculia executive dysfunction	–	Disorientation dyscalculia executive dysfunction	–	–
NPS	Depression anxiety	–	Behavioral changes anxiety	Anxiety irritation	Anxiety irritation
Myoclonus	–	–	–	–	–
Seizures	–	–	–	–	–
Pyramidal signs	–	–	–	–	–
Extrapyramidal signs	–	–	–	–	–
Cerebellar signs	–	–	–	–	–

*Key: MMSE, mini mental state examination; NPS, neuropsychiatric symptoms; +, present; –, not present; and NA, not available.*

## Discussion

In the present study, the *PSEN1* variant (c.529T > G, p.Phe177Val) was identified in a Chinese FAD family using WES. The variant was first reported in 2019 and classified as probably pathogenic according to the standard reported by Guerreiro et al. (2010) and Gao et al. (2019). However, the previous study did not perform co-segregation analysis or functional study. Furthermore, in the cell transfection studies, we found that the p.Phe177Val variant increased both the production of Aβ42 and the ratio of Aβ42 over Aβ40. Our results were consistent with findings regarding the p.Phe177Leu mutation in previous studies (Bai et al., 2015; Sun et al., 2017), and we further determined the p.Phe177Val variant of *PSEN1* as a pathogenic variant according to the ACMG standards. The p.Phe177Val variant is located in the third transmembrane domain (TM3) of the PSEN1 protein. The phenylalanine exchange resulting from the mutations may cause structural changes in the protein. The variant p.Phe177Val, in which the phenylalanine substituted for a valine, probably decreases the interaction with other hydrophobic residues and causes structural impairment (Gao et al., 2019). Another variant p.Phe177Ser, in which the phenylalanine is replaced by a serine, likely disrupts the tertiary structure of TM3 in the PSEN1 protein (Hausner et al., 2014). Furthermore, the variant p.Phe177Leu may impair the PSEN1 protein by reducing hydrophobic membrane association and protein stability (Somavarapu and Kepp, 2016).

Generally, the phenotype of FAD involving *PSEN1* mutations has been associated with early onset and rapid progression (Jia et al., 2020). Moreover, in addition to amnesia, some atypical clinical manifestations have been observed, such as behavioral changes, extrapyramidal signs, seizures, and myoclonus (Ryan et al., 2016). It has been previously reported that atypical phenotypes were more common in FAD patients with *PSEN1* post-codon 200 mutations (Tang et al., 2016). The proband with the p.Phe177Val variant was affected at the age of 45 and presented with amnesia, executive dysfunction, disorientation, and dyscalculia in a previous study (Gao et al., 2019). However, other than the typical cognitive symptoms, FAD patients with the same variant exhibited earlier onset, and neuropsychiatric symptoms in our study. Variants p.Phe177Ser and p.Phe177Leu, which affect the same amino acid, were first reported in a study screening for *PSEN1* mutations in patients with AD (Rogaeva et al., 2001), but no clinical features were described in that study. Two other reports have described the clinical characteristics of patients carrying a mutation affecting the same amino acid (p.Phe177Ser). Patients carrying this mutation in a German family with FAD presented with early onset (between 29 to 31 years of age) and rapid progression of memory decline (Hausner et al., 2014). Non-cognitive features were also observed in the patients, such as myoclonus, seizures, and pyramidal signs. In addition, the proband with p.Phe177Ser mutation in a Chinese family with FAD was affected by memory loss at the age of 30 and also demonstrated cerebellar ataxia and involuntary movement (Jiang et al., 2019). Another study reported that the p.Phe177Leu mutation is associated with epilepsy (Zarea et al., 2016). French patients with FAD carrying the p.Phe177Leu mutation demonstrated early onset (between 36 to 42 years), without atypical presentations (Raux et al., 2005). The differences in atypical phenotypes among the patients carrying different mutations at the same amino acid position suggested phenotypic heterogeneity among different FAD cases.

## Conclusion

In conclusion, we identify *PSEN1* p.Phe177Val variant in a Chinese FAD family across three generations using WES and confirm the pathogenicity of the variant in FAD. Furthermore, we broaden the clinical phenotype spectrum of FAD patients with *PSEN1* p.Phe177Val variant.

## Data Availability Statement

The raw data supporting the conclusions of this article will be made available by the authors, without undue reservation, to any qualified researcher.

## Ethics Statement

The studies involving human participants were reviewed and approved by Ethical approval was provided by the Research Committee of First Affiliated Hospital of Xiamen University. The patients/participants provided their written informed consent to participate in this study. Written informed consent was obtained from the individual(s) for the publication of any potentially identifiable images or data included in this article.

## Author Contributions

BJ and MB designed study, analyzed data, and drafted the manuscript. JL, QL, and N-AX contributed to cellular data acquisition and analysis. JF, M-YS, and Z-WY contributed to the clinical evaluation and specimen collection. Q-LM, S-JT, and K-MZ contributed to study design and funding acquisition. All authors contributed to the article and approved the submitted version.

## Conflict of Interest

The authors declare that the research was conducted in the absence of any commercial or financial relationships that could be construed as a potential conflict of interest.

## Footnotes

1 https://www.ncbi.nlm.nih.gov/variation/tools/1000genomes/

2 http://gnomad-old.broadinstitute.org/

3 https://exac.broadinstitute.org/

4 https://www.ncbi.nlm.nih.gov/snp/

5 http://sift.jcvi.org/

6 http://cadd.gs.washington.edu/

7 http://genetics.bwh.harvard.edu/pph2/
